# An Automated Bioinformatics Pipeline Informing Near-Real-Time Public Health Responses to New HIV Diagnoses in a Statewide HIV Epidemic

**DOI:** 10.3390/v15030737

**Published:** 2023-03-13

**Authors:** Mark Howison, Fizza S. Gillani, Vlad Novitsky, Jon A. Steingrimsson, John Fulton, Thomas Bertrand, Katharine Howe, Anna Civitarese, Lila Bhattarai, Meghan MacAskill, Guillermo Ronquillo, Joel Hague, Casey W. Dunn, Utpala Bandy, Joseph W. Hogan, Rami Kantor

**Affiliations:** 1 Research Improving People’s Lives, Providence, RI 02903, USA; 2 Department of Medicine, Brown University, Providence, RI 02906, USA; 3 Department of Biostatistics, Brown University School of Public Health, Providence, RI 02903, USA; 4 Department of Behavioral and Social Sciences, Brown University, Providence, RI 02903, USA; 5 Rhode Island Department of Health, Providence, RI 02908, USA; 6 Department of Ecology and Evolutionary Biology, Yale University, New Haven, CT 06511, USA

**Keywords:** molecular HIV clusters, phylogenetics, molecular epidemiology, HIV transmission networks, contact tracing, near-real-time data integration

## Abstract

Molecular HIV cluster data can guide public health responses towards ending the HIV epidemic. Currently, real-time data integration, analysis, and interpretation are challenging, leading to a delayed public health response. We present a comprehensive methodology for addressing these challenges through data integration, analysis, and reporting. We integrated heterogeneous data sources across systems and developed an open-source, automatic bioinformatics pipeline that provides molecular HIV cluster data to inform public health responses to new statewide HIV-1 diagnoses, overcoming data management, computational, and analytical challenges. We demonstrate implementation of this pipeline in a statewide HIV epidemic and use it to compare the impact of specific phylogenetic and distance-only methods and datasets on molecular HIV cluster analyses. The pipeline was applied to 18 monthly datasets generated between January 2020 and June 2022 in Rhode Island, USA, that provide statewide molecular HIV data to support routine public health case management by a multi-disciplinary team. The resulting cluster analyses and near-real-time reporting guided public health actions in 37 phylogenetically clustered cases out of 57 new HIV-1 diagnoses. Of the 37, only 21 (57%) clustered by distance-only methods. Through a unique academic-public health partnership, an automated open-source pipeline was developed and applied to prospective, routine analysis of statewide molecular HIV data in near-real-time. This collaboration informed public health actions to optimize disruption of HIV transmission.

## 1. Background

The HIV epidemic is an ongoing global public health burden and novel methods are needed to disrupt transmission. Analyzing genomic HIV data to guide public health response is one of the four pillars to end the US epidemic [1]. HIV-1 sequences routinely collected during clinical care for drug resistance testing can also be used to phylogenetically estimate viral evolution across individuals [2].

The real HIV transmission network among individuals is unknown. Public health agencies often engage in contact tracing to establish social networks among recently diagnosed individuals in order to identify and reach social contacts who may be infected but are undiagnosed [3]. Even incomplete social network data serves as a proxy for the real transmission network, providing relevant information to guide public health response. Similarly, phylogenetic relationships among sequences provide another independent source of information about social networks towards identifying undiagnosed or diagnosed-out-of-care individuals [4,5].

While much research has focused on phylogenies of molecular HIV data in academic settings [6], often long after sequenced individuals were diagnosed with HIV, there has been little investigation of the “real-time” employment of phylogenetics to assist with a public health response to newly diagnosed cases [7]. Poon et al. provided such information to British Columbia public health officials in near-real-time via automated phylogenetic analyses and monthly reports to public health, detecting and halting a transmitted drug resistance outbreak [8].

The paucity of such studies is likely due to challenges of integrating phylogenetic analysis into routine public health response, particularly in real-time [9]. First, phylogenetic analyses employ computationally expensive optimization methods, such as maximum likelihood and Bayesian inference, to search over a vast combinatorial space of possible phylogenies. Implementing these methods typically requires specialized computing clusters that are not readily available to public health agencies and analyses can take days or weeks to converge. Second, the availability of raw sequence data for analysis, longitudinally and in near-real-time, requires robust data management processes and expertise. Third, results of phylogenetic analysis (typically trees, confidence metrics, and sequence clustering) by themselves do not imply any specific action and must be analyzed, reported, integrated, and interpreted to guide a public health response, also in near-real-time. Finally, it is unclear whether phylogenetic analysis is necessary or whether computationally cheaper approaches based only on genetic distance are sufficient to inform a public health response.

This paper addresses these challenges through demonstration of an automated data management and bioinformatics pipeline, which has augmented the public health response to new HIV diagnoses in Rhode Island (RI). The pipeline is the result of a unique multidisciplinary partnership between the RI Department of Health (RIDOH) and academic researchers, who developed and used these tools in near-real-time to facilitate routine collaboration, case management, and a prospective study of re-interviewing newly HIV-diagnosed individuals who are members of molecular clusters [9].

## 2. Methods

The automated pipeline development steps included identification and curation of statewide databases, sequence quality control (QC) and analysis, datasets integration, summary reports, and data use for routine case management to inform near-real-time public health actions.

### 2.1. State-Wide HIV Databases

To integrate molecular HIV data with public health activities, three databases were collated and combined: (1) clinical database; (2) sequence database; (3) several public health databases.

The main source of *clinical data* is from The Miriam Hospital Immunology Center in Providence, RI, and its associated Immunology Center Database (ICDB). The Brown University Medical School affiliated Immunology Center is the largest HIV center in the state, providing comprehensive care for most (>80%) people with HIV, approximately 2000 upon this writing. The ICDB was created in 2003 for patient monitoring, Ryan White program reporting, and research. Since 2003, it evolved from manual data entry to integration with electronic medical records. Currently, HIV-specific data collection modules are created within Epic (the electronic medical record system) to download demographic, clinical, and laboratory data on Immunology Center patients.

The *sequence database* has curated all HIV-1 *pol* sequences from commercial laboratories conducting clinical care resistance testing at the Center since 2003. This prospective process has been augmented with monthly additions of non-Immunology Center sequences to complete a state-wide dataset that includes newly diagnosed and sequenced individuals.

The *public health databases* are maintained by RIDOH and contain contact tracing records, available sequence data, and laboratory results for all patients in the state, including those not treated at the Immunology Center.

### 2.2. Sequence QC and Analyses

Once newly generated, statewide, monthly sequences become available, sequences are analyzed by SQUAT principles [10] using the Sierra web service of the Stanford University HIV Drug Resistance Database [11] to identify sequences that contain >5% stop codons, G-to-A hypermutation, unusual mutations, and exact edit pairwise distance (0 when sites share the same nucleotides or ambiguities, or 1 otherwise) among new sequences and between them and historical sequences. Potentially problematic sequences and those of the same subtypes and <0.5% edit distance were flagged for further examination. HIV-1 subtype and drug resistance mutations were also identified in this process. Since all historical sequences were previously reviewed, only newly added sequences are included in the monthly QC report.

Once new sequence datasets pass QC, the pipeline identifies whether the most recently added sequences from new index cases are in molecular clusters. For this purpose, index cases are defined as HIV RI diagnoses within the past 6 months with an available new sequence. The pipeline performs multiple sequence alignment of the earliest single sequence per patient using MAFFT (version 7.313) [12]. It trims sites with 98% or more gaps in alignment using a custom R script. It also adds four HIV-1 group O sequences (GenBank accession numbers L20587, L20571, AY169812, AJ302647) to the alignment as outgroups in phylogenetic analyses.

For inclusivity, and since there is no consensus on which molecular epidemiology methods should be used, the pipeline performs phylogenetic analyses using several methods and parameters. The phylogenetic and clustering methods we used were based on a comprehensive comparative study, which is discussed in detail in prior work [13]. For brevity, we summarized the resulting methods from the comparative study, in which we compared both “strict” and “relaxed” cluster definitions. Here, we implemented relaxed parameters to favor false positive over false negative clusters and maximize available information. The pipeline implements the following five phylogenetic methods and cluster-defining parameters (bootstrap support and pairwise distance thresholds) from our comparative study: RAxML [14] (version 8.2.12; 80% bootstrap support; 4.5% genetic distance), IQ-TREE [15] in ultrafast bootstrap mode (version 2.0.4; 95% bootstrap support; 3.0% genetic distance), FastTree [16] (version 2.1.10; 80% bootstrap support; 4.5% genetic distance), FastTree with the alternative likelihood ratio test (version 2.1.10; 90% aLRT support; 3.0% genetic distance), and MEGA [17] (version 10.1.8; maximum-likelihood method; 80% bootstrap support; 4.5% genetic distance). Clusters are identified according to these parameters using ClusterPicker (version 1.2.3) [18].

In addition to phylogenetic analysis, the pipeline performs distance-only sequence clustering using HIV-TRACE (version 0.4.4) with the following two distance parameters: [19]. (1) 0.5%, recommended by the U.S. Centers for Disease Control and Prevention (CDC) [20] and by the HIV-TRACE authors for rapidly growing clusters [19]; and (2) 1.5%, determined by HIV-TRACE authors as best supported by previous research on the relationship between genetic distance and epidemiologically connected transmissions [19]. The distance parameter of 1.5% is calibrated with the findings from our previous comparative study that HIV-TRACE at a 1.5% distance threshold identified a comparable number of overall clusters as the phylogenetic methods and parameters described above [13]. The pipeline also identified CDC-defined “clusters of concern,” as HIV-TRACE clusters with a maximum 0.5% pairwise distance and three or more individuals diagnosed in the previous 12 months [21].

Lastly, in addition to comparing clustering methods, the pipeline compared clustering between a statewide dataset and an Immunology-Center-only subset, assessing the impact of an increased sampling density.

### 2.3. Data Integration

Once sequence analyses were completed, data from the three database sources (clinical, sequence, and public health) were joined with anonymized identifiers into a single integrated dataset. This dataset contains statewide sequences, demographics (e.g., gender, sex at birth, race, ethnicity, country of birth, home zip code), HIV diagnosis and last-negative dates, HIV risk factors, clinical data (e.g., illegal substance use, mental illness), laboratory data (e.g., CD4 and viral load), and contact tracing information (e.g., interview dates and numbers of named partners). The integrated dataset is cumulatively aggregated over time.

### 2.4. Report Generation

Each pipeline component automatically generates reports. After the QC reports detailed above, the phylogenetic analyses generate individual-level reports, summarizing clustering, demographics, and clinical information of newly generated sequences. For each index case sequence that clusters, a detailed report provides the phylogenetic clade containing the cluster and the most recent ancestor node alongside a summary of demographics and clinical information for cluster members. This visualization is implemented with custom R scripts using the ggtree and treeio packages [22,23]. A population-level report is also generated, providing statewide-level clustering summaries with a visualization of cluster growth over time, showing cluster membership of new and prior index cases.

### 2.5. Case Management

The pipeline is run monthly as new sequences are obtained and reports are generated for routine case management discussions between the RIDOH and academic partners, including clinicians, epidemiologists, disease intervention specialists, bioinformaticians, data managers, statisticians, virologists, evolutionary biologists, and public health staff. The pipeline reports guide discussions of each index case that is part of a molecular cluster by any of the five phylogenetic methods or HIV-TRACE, using either the statewide or Immunology Center dataset. This inclusive ensemble approach allows for comparison of clustering methods while minimizing false negatives and ensuring inclusion in public health interventions of cases with evidence of clustering by any method. The prospective evaluation of this intervention is ongoing; see details in a published study design [9].

The pipeline is available in an open-source software package from https://github.com/kantorlab/hiv-real-time-phylogeny (accessed on 12 March 2023). This study was approved by, and a consent waiver was obtained from, the Institutional Review Board at The Miriam Hospital, Providence, RI; and the RIDOH.

## 3. Results

### 3.1. Database and Sequence QC

At the 18th and latest pipeline analysis, the statewide dataset included 4290 *pol* sequences from 2440 patients. Of the 4290 sequences in the statewide dataset, 4140 sequences from 2316 patients were part of the Immunology Center subset. Thus, the statewide dataset added 150 sequences for 124 individuals. Of those 124, 53 were cared for in RI outside of the Immunology Center.

The pipeline QC process before study initiation identified 51 sequence pairs with pairwise genetic distance <0.5% that were resolved through manual review, leading to one excluded sequence that was identical to another sequence and may have been misattributed. Upon this resolution, ongoing monthly QC processes explored and flagged any QC or distance issues. The manual review burden for these QC steps was relatively low, occurring in only two out of 18 datasets. No sequences were flagged for stop codon, hypermutation, or unusual mutation criteria, indicating an expected good sequence quality.

### 3.2. Bioinformatic Pipeline

In the latest pipeline run, both HIV-TRACE analyses ran for ~15 min, while the five phylogenetic methods required between 4 min (FastTree) and 54 h (MEGA). The pipeline had a peak usage of 72 concurrent computer cores with 216 GB total memory, representing 0.5% of the 13,688 cores available on the computer cluster. Two previously tested phylogenetic methods (PhyML and IQ-TREE with regular bootstrapping) were excluded because of excessive runtimes presenting challenges for near-real-time analyses.

Table 1 summarizes the monthly counts of new index cases in RI and compares numbers of clustered index cases by method and dataset. Overall, 37 (65%) of 57 new HIV cases were identified as clustered by any method. These cases were selected for intervention and discussed in case management meetings. Of 37 clustered cases, 21 (57%) were identified by HIV-TRACE and 16 (43%) were detected exclusively by. All clusters identified by HIV-TRACE were also identified by at least one phylogenetic method. Minor differences in counts occurred between the statewide and Immunology Center datasets in four of the five phylogenetic methods and in three of the 18 datasets (bold/green in Table 1). These discrepancies included five individuals, three identified exclusively by the statewide dataset and two identified exclusively by the Immunology Center subset.

### 3.3. Reports and Case Management

**Figure 1:**
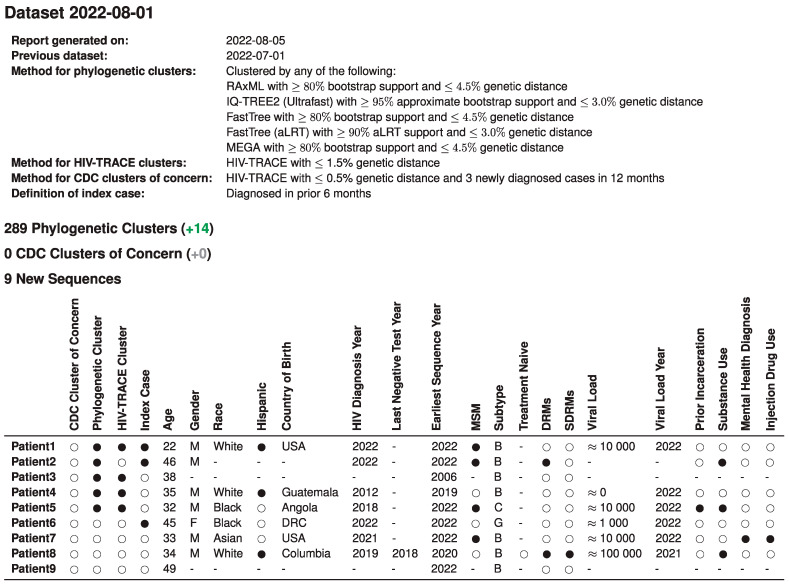
Title: Integrated report for monthly academic-public health case management meetings. Legend: An example of the summary page from automated monthly reports generated by the pipeline is illustrated here with synthetic data to protect patient privacy. The table summarizes demographic and clinical data of all nine newly available RI sequences in the past month, their index case status, and their cluster analysis outcomes that are relevant to the monthly case management meeting of new HIV diagnoses in the state. Numbers in green and gray in parentheses indicate comparison to the prior month. Missing values are indicated by ‘-’. Viral load is in copies/mL, approximated. MSM, men who have sex with men; DRMs, drug resistance mutations; SDRMs, surveillance drug resistance mutations. Notes: All information shown in this table is synthetic and for illustrative purposes only. No information from real patients is shown.

After dataset integration, individual and population reports were automatically generated and served as a basis for monthly case management discussions of the academic and public health teams. Figure 1 illustrates a summary report of monthly new sequences in a single table that displays integration of sequence, clinical, and public health data to guide case management. This initial report includes specific methods used, as well as information on the nine new sequences available that month. Of these nine, three fit the index case definition and of these, two were members of a phylogenetic cluster, and one was a distance-only cluster.

**Figure 2:**
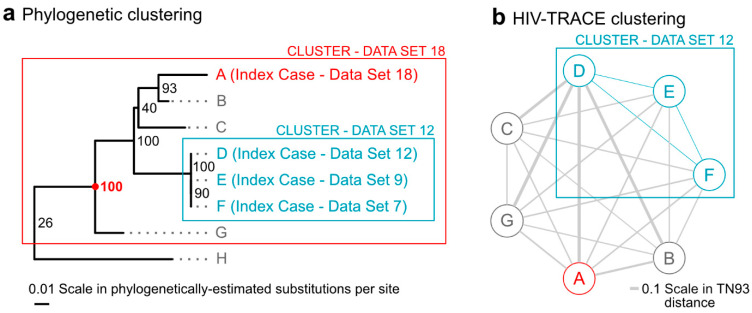
Title: A growing cluster with differential detection between phylogenetic and distance-only methods. Legend: The figure shows a comparison between a phylogenetic cluster (panel (**a**)) and a HIV-TRACE cluster graph (panel (**b**)) for those same cases. The 100% bootstrap supported (red 100) cluster in panel a (red box) contains a new index case from dataset 18 (A, red); three prior index cases from datasets 7, 9, and 12 (D–F, blue), which formed a cluster in the analysis of dataset 12 by both phylogenetic methods and HIV-TRACE at the 0.5% threshold; and previously un-clustered cases B, C, and G. Panel (**a**) also shows the nearest non-clustered case (H). The HIV-TRACE distance-based cluster graph in panel (**b**) of the same cases demonstrates that only cases D, E, and F remain part of the cluster in dataset 18 (blue edges). Cases A, B, C, and G are not clustered by HIV-TRACE as their pairwise distances (gray edges) are larger than distance thresholds established by the Centers for Disease Control and Prevention (CDC). Notes: Branch lengths in (**a**) are scaled by the estimated substitutions per site in the phylogeny, while edge thicknesses in (**b**) are scaled by the TN93 pairwise genetic distance calculated by HIV-TRACE. Phylogenetic bootstrap support (out of 100 bootstrap replicates) is shown in small text next to splits in the tree in (**a**) and the split with bootstrap support of 100 that defines the cluster is highlighted with a red dot.

Next, individual phylogenetic clusters of all new index cases who cluster are discussed and determinations regarding interventions are considered. Figure 2 shows an example of a specific growing cluster in the latest dataset, for which clustering differed between phylogenetic and distance-only methods. A cluster from the 12th dataset, which included index cases D, E, and F from datasets 12, 9, and 7, respectively, grows to a larger cluster in the 18th dataset by the addition of new index case A. This phylogenetic cluster, with 100% bootstrap support, now also includes cases B, C, and G. This cluster is identified by all phylogenetic methods but not by the distance-only method, since cases A, B, C, and G all have pairwise distances in HIV-TRACE that are above the 1.5% distance thresholds. Had it clustered by HIV-TRACE at the 0.5% distance threshold, the cluster would have met the criteria for a “cluster of concern” and would have been reportable to the CDC. In its current state, this cluster, which might indicate rapid growth and some public health concern, was identified by the pipeline and discussed in the case management meeting.

Population-level summary statistics allowing longitudinal overview of the RI epidemic demonstrated that 1176 (48%) of the 2440 individuals in the 18th statewide dataset were in molecular HIV clusters and seven individuals were in CDC clusters of concern.

**Figure 3:**
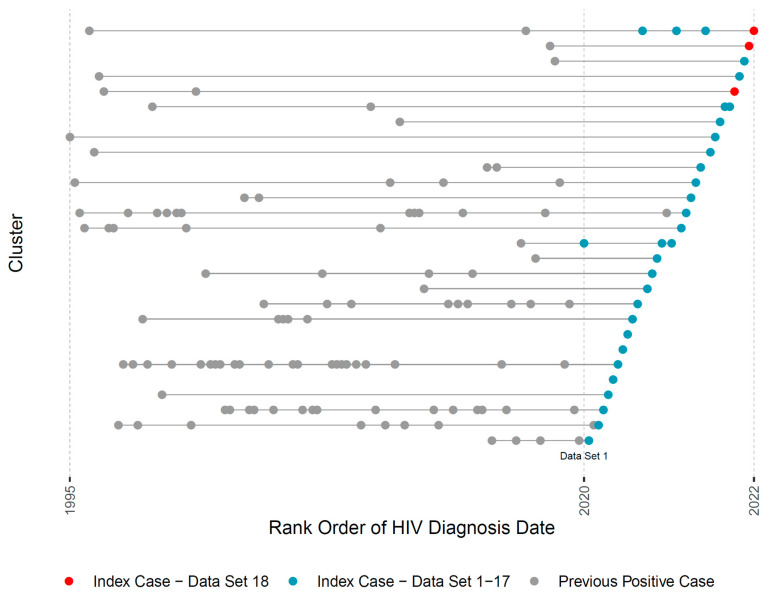
Title: Visual representation of HIV cluster growth in RI. Legend: This figure illustrates one visual output of the automated pipeline that is used in monthly academic-public health case management discussions. Each row represents one cluster’s lifespan according to years of HIV diagnosis of its members (X axis). Red dots indicate new index HIV cases in the current month. Blue dots indicate new HIV index cases in the prior 18 datasets. Gray dots indicate cluster members who were diagnosed prior to the start of the study. For patient privacy concerns, we use the rank ordering of diagnosis dates instead of the exact dates.

Lastly, the pipeline generates a visual summary of the evolving HIV molecular cluster membership in RI across monthly reporting (Figure 3; in the actual reports the X axis denotes exact diagnosis dates). Each horizontal line represents a cluster throughout its lifespan and dots represent individual members by their HIV-1 diagnosis date. Red dots represent current index cases that were individually discussed in the 18th (most recent) case management meeting. Blue dots represent index cases from prior months, allowing for visual exploration of cluster growth. Clusters containing both red and blue dots are of special concern since they may indicate active cluster growth, informing discussions and the urgency of public health intervention.

## 4. Discussion

The integration of phylogenetic analysis into a routine public health response to HIV is a complex, multi-step, and multi-disciplinary process. By assembling a diverse team of experts in public health, contact tracing, clinical care, infectious diseases, database management, sequence analysis, phylogenetics, statistics, and laboratory testing, we have developed and implemented an automated bioinformatics pipeline to analyze statewide HIV-1 sequence data prospectively and routinely. This process informs case management, guides public health interventions, and allows for prospective evaluation of the benefits of phylogenetic information in the design of public health interventions to disrupt HIV transmission in near-real-time.

Despite substantial recent progress in generating genomic data that informs clinical care, the timely and effective integration of heterogeneous data sources, such as genomic and clinical data, for use by health organizations remains a significant challenge [24]. Such data usually exist in separate databases and systems, managed by different agencies or organizations. A recent criticism has called for modernizing public health data and surveillance systems in the U.S. and facilitating better data sharing between health care organizations and public health agencies [25]. The pipeline we developed to integrate molecular epidemiology and traditional public health praxis in near-real-time is a small step towards the goal of using integrated heterogeneous data sources to improve health outcomes. An important prerequisite of this achievement is our cross-disciplinary team’s extensive efforts to aggregate and integrate RI data from clinical, sequence, and public health databases. Through our collaboration, we identified the relevant datasets and developed methods to enable their anonymous integration across systems. This process, which links wet laboratory, bioinformatics, analytical, and public health data, is essential to enable a statewide dataset that can be optimally used to disrupt HIV transmission.

Automation is essential for ensuring that analysis results are consistently prepared to meet the rapid pace of routine public health case management in accordance with end-the-HIV-epidemic concepts. Therefore, significant engineering efforts have gone into the development of the pipeline, which we have released as an open-source package for other public health teams to use and learn from. Our pipeline incorporates four features that, to our knowledge, are not available in previous approaches to automating HIV cluster analysis. First, we incorporated a flagging step prior to analysis to explore sequence quality. Second, we implemented multiple clustering methods, both phylogenetic and distance-only [13]. Third, we identified clustered individuals using an ensemble of these methods [9]. Fourth, we summarized clustering results using simple visual representations designed to facilitate routine real-time case management discussions with public health officials.

Other studies have investigated specific outbreaks in which cluster information was provided to public health officials. Examples include the 2015 outbreak of transmitted HIV drug resistance in British Columbia, Canada [8]; a 2015 HIV outbreak in Indiana, USA, linked to intravenous drug use that was investigated by the state health department and the CDC, where phylogenetic analysis guided contact tracing to identify 536 contacts among an initial cluster of 11 new HIV cases [26]; and a 2015–2016 CDC-led cluster analysis using distance-only methods that identified a growing cluster of new cases in Texas, USA, revealing a larger putative transmission network [27]. In contrast, the automated pipeline presented here allows such investigations to be performed and discussed routinely and in near-real-time. As clustering analyses become more widely used for HIV prevention, the usability and replicability of analytic methods and software will be increasingly important. Though traditionally distance-only methods are less computationally intensive and easier to use than phylogenetic methods, access to tools such as our automated pipeline might help lower the learning curve and remove obstacles to phylogenetic analysis, while also facilitating replicability.

In the comparison of clustering methods incorporated into the pipeline based on our prior work [13], we found that the ensemble of five commonly used phylogenetic methods identified 76% more clustered cases than the distance-only method alone. No clusters were identified solely by the distance-only method and phylogenetic methods alone were sufficient to identify all 37 new HIV cases selected for case management discussion. Based on these findings, it is reasonable to speculate that phylogenetic inference may be more beneficial than distance-based inference; however, caution should be advised in making such deductions, specific goals should be considered, and the benefits of each approach should be evaluated. Determining the public health benefits of identifying more clusters is a current need that we and others are actively addressing [9].

Since there is a single large clinic in the RI (the Immunology Center), we were able to study the difference in cluster identification between a clinic-based subset (which is simpler to assemble) versus a statewide dataset. The lack of major differences suggests that good sampling, approximately 80% in our case, of the state’s people living with HIV may provide sufficient data for overall cluster analysis. However, even in the small state of RI, of the additional 124 cases in the statewide dataset, 62 (50%) were part of clusters not observed in the Immunology Center subset. Although the size of RI may be a limitation in terms of assessing scalability of such findings, having a high, even if not full, statewide sequence sampling density is likely beneficial. How robust these findings are for larger jurisdictions and the eventual benefit of this improved cluster identification for public health remains to be seen [7]. Moreover, clustering is not always consistent and not all clusters are equal, as we recently reported [28]. Sequence addition can also result in reduction of clusters (e.g., dataset 14 where an additional cluster was detected in the Immunology Center subset relative to the statewide dataset), justifying careful interpretation and the need for longitudinal accumulation and analyses of cluster data.

Several potential limitations of our work and their implications should be noted. First, while the approach presented here works in the small state of RI, there may be logistical and scaling challenges with more sequences and longer analysis runtimes in a larger state or country. The availability of compute cores is less of an obstacle than the scalability of the phylogenetic algorithms themselves, as our analysis used only 0.5% of the capacity of the local compute cluster in RI, and national scientific computing centers have orders of magnitude greater compute capacity. Future work should investigate phylogenetic methods that reduce analysis time by, for example, using tools with less computation time (e.g., FastTree) or updating an existing phylogeny with new sequences instead of recalculating a new phylogeny. Related research on estimating “mega-phylogenies” for broadly sampled taxa and genes in evolutionary biology studies may hold insights for scaling HIV-1 phylogenies [29]. Second, as a small state, RI has been able to initiate contact tracing with every index case, which may not be feasible in larger jurisdictions where prioritization is essential [30]. The pipeline we have developed could help such prioritization policies and should be evaluated in such settings. Third, our data are only for new cases diagnosed within RI, even though infection may have occurred in another state or the individual may have moved out-of-state after diagnosis. Cluster analysis of out-of-state data might help improve the coordination of public health responses across jurisdictions. Fourth, the pipeline is currently designed for HIV-1 *pol* Sanger sequences obtained through routine clinical care. Enhancing the pipeline with “deeper” sequence data available through next-generation sequencing (NGS) technologies and exploring if and how the additional resolution of within-patient viral diversity improves cluster inference should be investigated [31]. Lastly, as in all studies of molecular HIV clusters, the true transmission networks among HIV cases are unknown, and in this regard, the limitations of this pipeline in evaluating which clustering method is best at recovering information from the true network should be recognized.

## 5. Conclusions

In an academic-public health partnership, to evaluate the incorporation of HIV molecular epidemiology into routine public health response, we have developed an open-source bioinformatics pipeline, overcoming challenges with data integration and management, computational scalability, analysis methodology, automation, and reporting. Applying this pipeline to 18 successive monthly datasets with newly diagnosed HIV cases informed routine case management in near-real-time and enabled realistic population- and individual-based representation of the statewide epidemic and its growth. Our multi-disciplinary approach facilitated evidence-based discussions and case management to disrupt HIV transmission in RI. Results from the pipeline have enabled an ongoing prospective study to evaluate the benefits of molecular epidemiology for planning public health responses to the ongoing and ever-challenging HIV epidemic [9], a priority of the U.S. Department of Health and Human Services [1].

## Figures and Tables

**Table 1:** Clustering of new HIV cases in RI by dataset number, dataset sampling density (statewide versus Immunology Center), and clustering method.

	Dataset Number																		
	1	2	3	4	5	6	7	8	9	10	11	12	13	14	15	16	17	18	Total
*Index Cases*	1	1	1	5	6	5	5	5	3	5	0	3	3	3	0	2	5	4	57
*Clustered in Statewide Dataset:*																			
Any Phylogenetic Method	1	1	1	2	5	2	4	1	2	2	0	3	2	3	0	2	3	3	37
RAxML	1	1	1	2	5	2	4	1	2	2	0	3	2	3	0	2	3	2	36
IQ-Tree	1	1	1	1	5	2	3	1	2	1	0	2	2	**1**	0	2	3	2	**30**
FastTree	1	1	1	1	4	2	4	1	2	**1**	0	3	**2**	2	0	2	3	3	**33**
FastTree (ALRT)	1	1	1	1	5	2	3	1	2	1	0	2	1	**1**	0	2	3	2	**29**
MEGA	1	1	1	2	5	2	4	1	2	1	0	3	**2**	2	0	2	3	1	**33**
HIV-TRACE (1.5%)	1	1	1	1	4	2	1	1	2	0	0	1	0	1	0	2	3	0	21
CDC Cluster of Concern (0.5%)	0	0	0	0	0	0	0	0	0	0	0	1	0	0	0	0	0	0	1
Only by Phylogenetic Methods	0	0	0	1	1	0	3	0	0	2	0	2	2	2	0	0	0	3	16
*Clustered in Immunology Center Subset:*																			
Any Phylogenetic Method	1	1	1	2	5	2	4	1	2	2	0	3	2	3	0	2	3	3	37
RAxML	1	1	1	2	5	2	4	1	2	2	0	3	2	3	0	2	3	2	36
IQ-Tree	1	1	1	1	5	2	3	1	2	1	0	2	2	**2**	0	2	3	2	**31**
FastTree	1	1	1	1	4	2	4	1	2	**0**	0	3	**1**	2	0	2	3	3	**31**
FastTree (ALRT)	1	1	1	1	5	2	3	1	2	1	0	2	1	**2**	0	2	3	2	**30**
MEGA	1	1	1	2	5	2	4	1	2	1	0	3	**1**	2	0	2	3	1	**32**
HIV-TRACE (1.5%)	1	1	1	1	4	2	1	1	2	0	0	1	0	1	0	2	3	0	21
CDC Cluster of Concern (0.5%)	0	0	0	0	0	0	0	0	0	0	0	1	0	0	0	0	0	0	1
Only by Phylogenetic Methods	0	0	0	1	1	0	3	0	0	2	0	2	2	2	0	0	0	3	16

Notes: Green cells are those that differ between the statewide datasets and the Immunology Center subsets.

## Data Availability

Data collected for this study contains Protected Health Information (PHI), accessed by agreement between Lifespan and the Rhode Island Department of Health, and cannot be made publicly available. Please contact Rami Kantor (rkantor@brown.edu) for data-related inquiries. The data dictionary and bioinformatics methods are available from https://github.com/kantorlab/hiv-real-time-phylogeny (accessed on 12 March 2023).
